# The skeletal muscle–adipose creatine metabolic axis: A novel paradigm for lipid metabolism reprogramming and obesity management

**DOI:** 10.1113/EP093049

**Published:** 2025-10-10

**Authors:** Yuhui Su, Na Liu, Yang Liu, Yiqun Sun, Yike Jiao

**Affiliations:** ^1^ College of Human Movement Science Jilin Sport University Changchun China; ^2^ College of Sports Rehabilition Beijing Sport University Beijing China

**Keywords:** creatine metabolism, exercise adaptation, futile creatine cycling, metabolic axis, white adipose tissue browning

## Abstract

The global prevalence of obesity and related metabolic disorders has spurred interdisciplinary research to develop new intervention strategies. Current research is increasingly focusing on the exercise‐induced browning of white adipose tissue and the mechanisms by which it improves energy metabolism. Creatine, as the primary carrier of high‐energy phosphate bonds within cells, is gaining attention for its role in the metabolic reprogramming of adipose tissue. This review aims to clarify the synergistic regulatory mechanisms between exercise and creatine metabolism, and introduces an innovative ‘skeletal muscle–adipose creatine metabolic axis’ model. Exercise may upregulate the expression of the creatine transporter in skeletal muscle by activating the AMP‐activated protein kinase/peroxisome proliferator‐activated receptor γ coactivator 1‐α signalling pathway, enhancing phosphocreatine shuttle kinetics, and thereby increasing energy metabolism efficiency. Concurrently, exercise‐induced exosomes or miRNAs from skeletal muscle may regulate the futile creatine cycle in adipose tissue and activate non‐uncoupling protein 1‐dependent thermogenic pathways, thus alleviating obesity conditions. This model not only reveals the multi‐organ cross‐talk mechanism mediated by exercise in lipid metabolism regulation but also provides a theoretical basis for creatine metabolism‐targeted obesity interventions.

## Abstract (highlights)

- **What is the topic of this review?**
  This review aims to clarify the synergistic regulatory mechanisms between exercise and creatine metabolism.
- **What advances does it highlight?**
  This review proposes an innovative ‘skeletal muscle–adipose creatine metabolic axis’ model, revealing how exercise and creatine synergistically enhance energy metabolism via AMPK/PGC‐1α signalling and exosome‐mediated inter‐tissue crosstalk.

## 1 INTRODUCTION

The global prevalence of obesity persists in escalating, with the World Obesity Federation forecasting that in excess of 1.1 billion adults will satisfy the criteria for obesity by the year 2030, concomitant with a marked increase in obesity‐related metabolic disorders, including type 2 diabetes and cardiovascular diseases (World Obesity Federation, 2025). This concerning progression highlights the pressing necessity for comprehensive interventions that address energy imbalance and systemic metabolic dysfunction. Exercise, as a viable non‐pharmacological approach, exhibits considerable anti‐obesity benefits by augmenting the plasticity of adipose tissue, particularly through the promotion of white adipose tissue (WAT) browning (Aldiss et al., 2018). Nevertheless, the molecular mechanisms governing this process are not fully understood. Recent breakthroughs in creatine metabolism research have expanded beyond its conventional role in high‐energy phosphate metabolism within muscle, elucidating its pivotal function in adipose thermogenesis and inter‐organ communication, thereby presenting new opportunities for the prevention and management of metabolic diseases.

Brown adipose tissue was initially recognized in the 16th century by the Swedish scientist Conrad Gessner; however, its physiological roles were not elucidated until the 1960s (Smith & Hock, 1963). Subsequent research has substantiated that white adipocytes can be converted into beige adipocytes via exposure to cold, hormonal agents, adipomyokines or physical exercise, thereby adopting a thermogenic profile akin to that of brown adipose tissue (Scheel et al., 2022; Sun et al., 2021). This transformation, referred to as WAT browning, wherein energy‐storing adipocytes acquire thermogenic capabilities, has been recognized as a crucial mechanism for augmenting energy expenditure (Kurylowicz & Puzianowska‐Kuznicka, 2020). While the thermogenic pathway reliant on uncoupling protein 1 (UCP1) has been the subject of extensive investigation (Anunciado‐Koza et al., 2008; Ikeda & Yamada, 2020), recent evidence underscores an alternative pathway that does not depend on UCP1, which is activated by futile creatine cycling within adipocytes. This process, involving the phosphorylation and deph70068osphorylation of creatine, converts chemical energy into heat, a mechanism that is notably amplified by cold exposure, β‐adrenergic stimulation or pharmacological treatments (Chouchani et al., 2019; Ikeda & Yamada, 2020). Notably, the genetic ablation of critical regulators in adipose creatine metabolism, such as glycine amidinotransferase (GATM; the rate‐limiting enzyme in creatine biosynthesis) or the creatine transporter (CRT), has been shown to suppress diet‐induced thermogenesis and exacerbate obesity in murine models (Kazak et al., 2017, 2019). These findings unequivocally establish adipose creatine metabolism as a central node in the regulation of energy homeostasis.

Skeletal muscle, acting as the primary reservoir for bodily creatine (accounting for approximately 95% of total stores), plays a dynamic role in regulating exercise‐induced metabolic adaptations. The creatine–phosphocreatine (Cr/PCr) shuttle system facilitates the long‐range transport of ATP to subcellular sites of demand and is crucial for rapid ATP regeneration during muscle contractions. Studies suggest that catecholamine hormones, such as noradrenaline, isoproterenol and clenbuterol, enhance skeletal muscle creatine uptake through cAMP‐dependent β_2_‐adrenergic receptor signalling (Odoom et al., 1996). Importantly, transcriptional and translational differences in the creatine transporter (CRT) across various skeletal muscle fibre types directly affect phosphocreatine shuttle activity (Brault & Terjung, 2003). In cardiomyocytes cultured in creatine‐depleted media or treated with the AMP‐activated protein kinase (AMPK) activator 5‐aminoimidazole‐4‐carboxamide ribonucleotide (AICAR), creatine transport is increased, correlating with changes in cell‐surface CRT abundance, indicating AMPK‐mediated regulation of creatine transport (Darrabie et al., 2011). The overexpression of *Pgc‐1α* or *Pgc‐1β* in L6 myotubes significantly increases *Crt* mRNA levels and creatine uptake (Brown et al., 2014), while chronic creatine supplementation decreases skeletal muscle CRT expression, suggesting feedback regulation between intracellular creatine levels and transporter activity (Guerrero‐Ontiveros & Wallimann, 1998). Beyond their traditional energy‐buffering function, skeletal muscle‐derived factors, including irisin, myostatin and exosomal miRNAs, have been demonstrated to suppress adipogenesis and promote WAT browning (Bostrom et al., 2012; Vechetti et al., 2021; Wang et al., 2022), highlighting skeletal muscle not only as an organ responsive to exercise but also as a central node regulating metabolic adaptation in remote adipose tissues.

**FIGURE 1:**
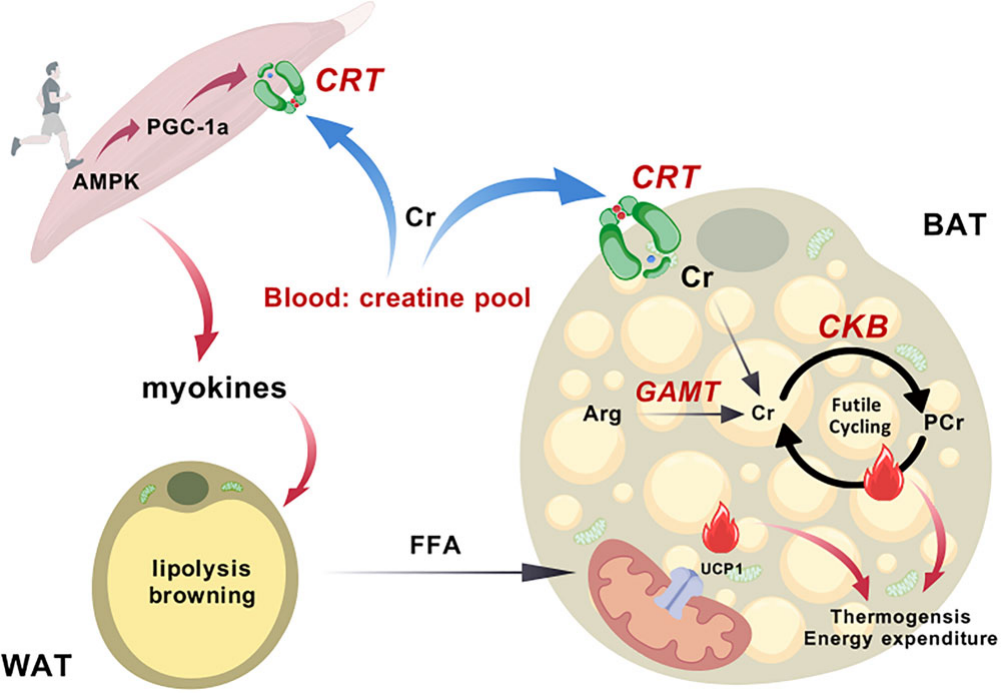
The skeletal muscle–adipose creatine axis. Exercise activates the AMP‐activated protein kinase (AMPK)/peroxisome proliferator‐activated receptor γ coactivator 1‐α (PGC‐1α) pathway in skeletal muscle (Lira et al., 2010), upregulating CRT activity and optimizing Cr/PCr shuttle kinetics, thereby potentiating endogenous creatine synthesis and glucose/lipid metabolic efficiency. In addition, exercise‐induced sympathetic activation or myokine secretion modulates futile creatine cycling in adipose tissue, activating non‐UCP1‐dependent thermogenesis. Crucially, key regulators such as AMPK and PGC‐1α coordinate CRT expression and mitochondrial creatine metabolism, thereby coupling muscle energy flux with adipose plasticity (Brown et al., 2014; Darrabie et al., 2011; Jager et al., 2007b). BAT, brown adipose tissue; CKB, creatine kinase B; CRT, creatine transporter; FFA, free fatty acids; GAMT, glycine amidinotransferase; UCP1, uncoupling protein 1.

This review proposes a concept of ‘skeletal muscle–adipose creatine metabolic axis’ to elucidate the synergistic interplay between creatine dynamics and adipose remodelling (Figure 1). The bidirectional crosstalk between skeletal muscle creatine metabolism and adipose thermogenesis represents a paradigm shift in understanding exercise‐mediated metabolic benefits. Deciphering the molecular dialogues within this axis may unveil novel therapeutic targets for obesity and its complications. This framework not only redefines current understanding but also lays the groundwork for integrated intervention strategies combining exercise regimens with creatine metabolism modulation.

## 2 ADIPOSE TISSUE HETEROGENEITY AND THE CREATINE METABOLIC NETWORK

### 2.1 Functional divergence and metabolic plasticity of adipose subtypes

Adipose tissue, as a highly heterogeneous endocrine organ (Kershaw & Flier (2004), can be classified into three main subtypes based on its developmental origin, morphological characteristics, and functional specialization: WAT, brown adipose tissue (BAT) and beige adipose tissue. WAT originates from mesodermal precursor cells and is widely distributed in subcutaneous and visceral regions. This tissue stores excess energy in lipid droplets; however, its pathological expansion, characterized by hypertrophy and dysfunction of adipocytes, is a key factor in inducing insulin resistance and chronic inflammation (Sun et al., 2021). BAT, derived from myogenic progenitor cells during embryonic development, contains multiple compartmentalized lipid droplets and abundant mitochondria within its cells. The hallmark protein UCP1 in BAT converts the proton gradient generated by mitochondrial respiratory chain through a proton leak mechanism into heat, significantly increasing energy expenditure during cold exposure or sympathetic activation (Chouchani et al., 2019). Beige adipose tissue exhibits unique plasticity: it is formed from precursor cells in WAT and activates thermogenic mechanisms dependent on UCP1 and independent of UCP1 under the influence of β_3_‐adrenergic receptor (β_3_‐AR) agonists, exercise, or peroxisome proliferator‐activated receptor γ (PPARγ) agonists (Bertholet et al., 2017). Single‐cell sequencing technology has revealed significant heterogeneity within beige adipocyte populations, identifying at least two functional subpopulations: a high UCP1 subpopulation that relies on classical thermogenic pathways; and a phosphocreatine metabolism dominant subpopulation that achieves non‐UCP1‐dependent thermogenesis through an inefficient phosphocreatine cycle (Kazak et al., 2015). This metabolic diversity provides the theoretical basis for therapeutic strategies targeting different adipose subtypes.

BAT exhibits superior thermogenic capacity compared to the liver and skeletal muscle (Rothwell & Stock (1983). While classical theories attribute adipose thermogenesis entirely to UCP1‐mediated mitochondrial proton leak – uncoupling fatty acid oxidation from ATP production to dissipate energy as heat (Wu et al., 2013) – emerging evidence challenges this paradigm. Notably, epididymal adipocytes lacking UCP1 display robust futile creatine cycling (Bertholet et al., 2017; Sun et al., 2021). Kazak et al. (2015) demonstrated that cold‐induced beige adipose mitochondria exhibit significant enrichment of arginine/creatine metabolic pathway proteins (e.g., GATM, mitochondrial creatine kinase 2 (CKMT2)), with futile creatine cycling contributing up to 30% of thermogenic capacity independently of UCP1. This cycle consumes ATP through repetitive phosphorylation (Cr → PCr) and dephosphorylation (PCr → Cr), releasing chemical energy as heat. Genetic evidence corroborates this mechanism: adipose‐specific knockout of creatine kinase B (*Ckb*) reduces diet‐induced thermogenesis (DIT), exacerbates obesity and impairs glucose homeostasis (Rahbani et al., 2021). Similarly, adipose‐specific deletion of the creatine transporter (*Crt*/*Slc6a8*) markedly suppresses DIT, promoting fat accumulation and obesity in high‐fat diet‐fed mice (Kazak et al., 2019). Intriguingly, creatine cycling synergizes with UCP1 pathways: in *Ucp1* ^−/−^ mice, cold exposure upregulates creatine metabolism genes (e.g., *Gatm*, *Ckb*), partially compensating for thermogenic deficits (Sun et al., 2021). Sun et al. (2021) further demonstrated that ablating mitochondrial alkaline phosphatase (TNAP) in adipocytes – which catalyses PCr hydrolysis to drive futile cycling – reduces energy expenditure and exacerbates diet‐induced obesity in mice. This compensatory plasticity suggests that targeting creatine cycling may offer alternative therapeutic strategies for obesity patients with low UCP1 activity. Despite containing minimal creatine reserves, adipose tissue leverages its creatine metabolic network – via the unique ‘futile cycling’ mechanism – to establish itself as a critical thermogenic pathway beyond UCP1 (Kazak & Cohen (2020).

### 2.2 Creatine cycling: A secondary engine of adipose thermogenesis

#### 2.2.1 The GATM‐driven thermogenic engine

Kazak et al. (2017) revealed the critical role of creatine metabolism in adipocytes in energy expenditure and obesity regulation by constructing a specific fat tissue *Gatm* gene knockout mouse model (Adipo‐Gatm KO). This provides a new theoretical perspective for metabolic adaptation mechanisms. By using the adiponectin–Cre system to specifically delete the *Gatm* gene in adipocytes, researchers successfully reduced the expression of GATM protein in BAT and subcutaneous adipose tissue, and observed a significant decrease in creatine and phosphocreatine levels in BAT. Liquid chromatography–mass spectrometry (LC‐MS) metabolomite analysis further confirmed that the absence of the *Gatm* gene specifically inhibited the production of downstream metabolic products of creatine synthesis (such as creatine and phosphocreatine), while upstream metabolic products (such as arginine and glycine) were unaffected. The compensatory upregulation of UCP1 protein in subcutaneous adipose tissue revealed a functional interaction between creatine metabolism and UCP1‐mediated thermogenic function. Adipo‐Gatm KO mice showed a significant drop in core body temperature under acute cold exposure (4°C) and rapidly developed obesity under high‐fat diet (HFD) conditions, with fat mass increasing by 80–85%, and metabolic efficiency (weight/fat increase per calorie intake) increased. Despite comparable energy intake to the control group, KO mice exhibited impaired glucose tolerance and fasting hyperinsulinaemia, indicating early metabolic dysfunction (Kazak et al., 2017). This study first provided genetic evidence proving that creatine metabolism in adipose tissue is a key effector pathway for DIT.

Although *Ucp1* KO mice maintained energy balance through compensatory mechanisms, irreversible DIT damage in *Gatm* gene knockout mice exacerbated obesity, highlighting the unique role of creatine metabolism in metabolic adaptation. Human studies also confirmed these findings: BAT highly expresses mitochondrial creatine kinase (CKMT1/2), and creatine regulation significantly affects oxidative metabolism in human adipocytes (Kazak et al., 2015), indicating the evolutionary conservation of this pathway. Future research should further explore the synergistic effects between creatine metabolism and UCP1 and investigate dietary or drug strategies to enhance ineffective creatine cycles as potential means for obesity intervention.

#### 2.2.2 The CRT‐driven thermogenic engine

The role of adipocyte creatine metabolism in energy balance and obesity regulation has garnered increasing attention. Recent studies reveal that functional impairment of the creatine transporter (CrT/Slc6a8) in adipose tissue profoundly disrupts thermogenesis and energy metabolism. Kazak et al. (2019) systematically deciphered the mechanistic role of creatine transport in adaptive thermogenesis and obesity pathogenesis using adipose‐specific *CrT* knockout mice (*AdCrTKO*). Their findings demonstrated that *AdCrTKO* mice exhibited ∼60% and ∼40% reductions in creatine and PCr levels in BAT and subcutaneous adipose tissue (SQ), respectively, alongside a 10–15% decrease in whole‐body energy expenditure induced by the β_3_‐adrenergic agonist CL 316,243. This metabolic defect was exacerbated under HFD conditions: *AdCrTKO* mice displayed 50% and 37% increases in metabolic efficiency at 30°C and 22°C, respectively, with marked fat mass accumulation, elevated triglyceride (TG) content in BAT and SQ, and a twofold increase in adiposity in *Crt* ^−/y^ mice (a high‐fidelity model of human CRT deficiency; Perna et al., 2016), indicating that impaired creatine‐dependent thermogenesis exacerbates energy storage propensity (Kazak et al., 2019).

Blocking adipocyte creatine transport blunts the sensitivity of diet‐ and β_3_‐adrenergic‐induced thermogenic responses, restricts adipose browning, reduces systemic energy expenditure and thereby promotes obesity. Clinically, subcutaneous adipocyte *CRT* expression inversely correlates with body mass index and positively associates with insulin sensitivity in humans (Kazak et al., 2019). These observations suggest that enhancing adipocyte creatine transport may represent a viable strategy to boost energy expenditure and combat obesity.

#### 2.2.3 The creatine kinase B‐driven thermogenic engine

Rahbani and his colleagues revealed the core role of creatine kinase B (CKB) in thermogenesis by the futile creatine cycle in brown adipocytes. Researchers used stable isotope tracing techniques combined with LC‐MS/MS to detect significant creatine kinase activity within brown adipocytes, with CKB being the predominant isoform. Specific knockout of the *Ckb* gene in mice showed reduced thermogenic capacity of brown adipocytes, increased susceptibility to obesity, and disrupted glucose homeostasis, confirming the crucial role of CKB in thermogenesis in brown adipose tissue. Further experiments indicated that CKB expression is regulated by the cAMP signalling pathway and is activated under cold exposure or β_3_‐adrenergic receptor stimulation. Notably, CKB is partially localized to the mitochondria of brown adipocytes, with its mitochondrial targeting sequence (iMTS‐L) being important for subcellular localization. Both in vivo and in vitro experiments confirmed that mitochondrial CKB catalyses the phosphorylation of creatine to release ADP, promoting mitochondrial respiration. In the futile cycle, during the phosphorylation phase, CKB phosphorylates creatine to generate PCr and releases ADP; during the hydrolysis phase, PCr hydrolyses back to creatine, releasing energy to synthesize ATP and maintain mitochondrial respiration. This ATP‐independent cyclic mechanism allows brown adipocytes to increase ADP flux, amplify energy dissipation, enhance mitochondrial uncoupling and improve thermogenic efficiency (Rahbani et al., 2021).

#### 2.2.4 The amplifier role of TNAP

Sun et al. (2021) identified tissue‐non‐specific alkaline phosphatase (TNAP) as a critical regulator of futile creatine cycling in brown adipocytes through its hydrolysis of PCr. Using ^31^P‐nuclear magnetic resonance, they detected robust PCr phosphatase activity in mitochondrial protein extracts from BAT and inguinal WAT (iWAT) containing beige adipocytes. This activity was markedly enhanced under cold exposure, indicating its thermogenic relevance. Biochemical fractionation and mass spectrometry analyses revealed TNAP as the predominant contributor to this PCr phosphatase activity. TNAP expression was induced in brown adipocytes and further upregulated by cold stimuli. Intriguingly, TNAP exhibited atypical mitochondrial localization in brown adipocytes – distinct from its canonical cell‐surface or secreted forms in other cell types. Immunofluorescence and electron microscopy confirmed TNAP's mitochondrial targeting, with predominant distribution in the intermembrane space and inner mitochondrial membrane, a spatial arrangement critical for its thermogenic function. To validate TNAP's role in futile cycling, treatment of brown adipocyte mitochondria with the TNAP inhibitor SBI‐425 significantly suppressed creatine‐dependent oxygen consumption, demonstrating TNAP's essentiality in sustaining this cycle. Rapid mitochondrial extraction and metabolite profiling further revealed that TNAP inhibition caused pronounced PCr accumulation in mitochondria, directly confirming its enzymatic role in PCr hydrolysis. in vivo studies showed that SBI‐425‐treated mice exhibited reduced whole‐body energy expenditure following β_3_‐adrenergic stimulation with CL 316,243. Adipocyte‐specific *Alpl* (TNAP‐encoding gene) knockout (*Adipo‐Alpl* KO) mice displayed accelerated weight gain and fat mass accumulation under HFD feeding, solidifying TNAP's pivotal role in energy expenditure regulation and obesity prevention (Sun et al., 2021).

## 3 EXERCISE‐INDUCED ADIPOSE REMODELLING: COORDINATED REGULATION OF THE SKELETAL MUSCLE–ADIPOSE CREATINE METABOLIC AXIS

### 3.1 Effects of exercise and creatine supplementation on fat mass

The effects of creatine supplementation combined with resistance training on human body composition have been extensively studied across different age groups. A 2019 meta‐analysis in adults ≥50 years old showed that creatine supplementation significantly reduced body fat percentage (−0.55%, *P* = 0.04), and although the reduction in absolute fat mass did not reach statistical significance (−0.50 kg, *P* = 0.13), the change may still be clinically relevant (Forbes et al., 2019). A 2023 meta‐analysis in adults <50 years old found that creatine supplementation significantly decreased body fat percentage (−1.19%, *P* = 0.006) but did not reduce absolute fat mass (−0.18 kg, *P* = 0.76) (Candow et al., 2023). Researchers suggest that the decrease in body fat percentage may be attributed to increases in lean mass rather than direct fat loss. Mechanistic studies indicate that creatine may indirectly modulate body composition by influencing adipocyte bioenergetics, inhibiting triglyceride synthesis and enhancing resting energy expenditure. Overall, creatine supplementation does not increase fat mass and may slightly improve body fat percentage, particularly in the context of resistance training.

### 3.2 Exercise promotes adipose tissue remodelling via sympathetic nervous system activation

Exercise activates the sympathetic nervous system (SNS), accelerating lipolysis, enhancing skeletal muscle energy utilization and expenditure, and thereby reducing adipose deposition (Martin et al., 2020). The SNS and noradrenergic postganglionic neurons maintain the development of brown phenotypes in adipose tissue through β‐adrenergic receptor signalling (Bachman et al., 2002). The density of noradrenergic fibres in adipose depots is positively correlated with the functional activity and proportion of thermogenic adipocytes (Merlin et al., 2016). As a classical regulator of brown adipose activity (Zouhal et al., 2008), exercise stimulates sympathetic nerves, elevating levels of circulating noradrenaline, which binds to β‐adrenergic receptors on brown adipocytes to activate the cAMP‐dependent protein kinase A (PKA) signalling pathway. PKA activation promotes lipolysis, upregulates UCP1 expression, and enhances brown adipose activation and thermogenesis (Merlin et al., 2016).

Robust SNS activation is observed during exercise (Christensen & Galbo, 1983; Zouhal et al., 2008). Chronic endurance training increases adipose tissue β_3_‐AR density and potentiates catecholamine signalling, driving WAT toward a beige phenotype (De Matteis et al., 2013). A single bout of resistance exercise acutely elevates circulating stress‐related hormones (noradrenaline and corticosterone) and muscle phosphorylation levels of AMPK, p38 mitogen‐activated protein kinase and cAMP response element‐binding protein (Abdalla‐Silva et al., 2024). AMPK‐mediated phosphorylation of PGC‐1α initiates critical transcriptional regulation of genes involved in skeletal muscle metabolism (Jager et al., 2007a). Notably, the AMPK activator AICAR enhances cardiomyocyte creatine transport by increasing cell‐surface CRT abundance (Darrabie et al., 2011). Similarly, adenovirus‐mediated overexpression of *Pgc‐1α* or *Pgc‐1β* in L6 myotubes elevates *Crt* mRNA levels and creatine uptake (Brown et al., 2014). Together, exercise activates the AMPK/PGC‐1α axis in myocytes via SNS stimulation, upregulating CRT expression to enhance creatine uptake, amplify skeletal muscle energy expenditure and promote WAT browning, collectively counteracting obesity.

### 3.3 Skeletal muscle‐derived myokines as endocrine mediators of adipose tissue remodelling

Skeletal muscle serves as a core hub for glucose and lipid metabolism and endocrine regulation, secreting various myokines through autocrine or paracrine mechanisms (Pedersen & Febbraio, 2012). These myokines facilitate dynamic inter‐organ metabolic communication, which is crucial for maintaining whole‐body homeostasis. Within skeletal muscle, myocytes interact with intramuscular adipocytes via cytokines and exosomes. In 2012, Bruce Spiegelman's groundbreaking research revealed that overexpressing PGC‐1α in skeletal muscle leads to secretion of fibronectin type III domain‐containing protein 5 (FNDC5), which, after proteolytic cleavage, releases the irisin hormone, capable of inducing browning of white fat tissue and combating obesity (Bostrom et al., 2012). Numerous classical myokines play key roles in regulating signal exchange within the muscle–adipose axis (Fang et al., 2023; Pedersen & Febbraio, 2012). Besides irisin, classic myokines such as myostatin (MSTN) and meteorin‐like protein (Metrnl) inhibit preadipocyte differentiation and lipid accumulation in animal models (Schmid et al., 2021). Exosomes derived from myocytes containing interleukin (IL)‐1α, IL‐1β and IL‐15 mediate intercellular signalling, inhibiting adipogenesis and lipid droplet formation (Kong et al., 2018; Vumbaca et al., 2021).

Recent advancements in multi‐omics technologies have further identified new myokines, adipokines and exosomal miRNAs that coordinate muscle–adipose communication to regulate systemic metabolism (Guo et al., 2023). Exercise preferentially activates lipolytic myokines, promoting glycerol release and fatty acid oxidation to meet energy demands during physical activity. The classical exercise‐induced myokine IL‐6 upregulates UCP1 expression, driving inguinal white fat browning (Knudsen et al., 2014). Serum from exercised mice or C2C12 myotubes treated with AMPK agonists can enhance UCP1 expression and thermogenesis in adipocytes (Kim et al., 2022). Exercise also elevates circulating levels of irisin, MSTN, fibroblast growth factor 21 (FGF21) and Metrnl, which upregulate genes associated with brown fat cells (such as *UCP1*), improving energy expenditure and thermogenesis both in vivo and in vitro (Li et al., 2019; Pervin et al., 2021; Rao et al., 2014). Notably, exercise‐derived exosomal miR‐27a promotes WAT browning by targeting PPARγ and activates the insulin receptor substrate 1 (IRS1)–Akt–glucose transporter type 4 (GLUT4) signalling axis, mitigating obesity induced by high‐fat diet (Wang et al., 2022).

## 4 CLINICAL TRANSLATION OF THE SKELETAL MUSCLE–ADIPOSE CREATINE AXIS: FROM MECHANISTIC INSIGHTS TO PRECISION INTERVENTIONS

The skeletal muscle–adipose creatine axis provides a new dimension for the treatment of obesity and related metabolic disorders. By integrating molecular regulatory networks, cross‐tissue metabolic crosstalk and clinical evidence, this section systematically evaluates the translational potential, current challenges, and breakthrough strategies along this axis. Targeted interventions on the creatine metabolic network demonstrate the transformative potential for obesity management. Key priorities include elucidating the mechanisms of creatine transport between tissues, developing spatiotemporally precise intervention measures, and validating their clinical efficacy through randomized controlled trials.

For example, selective creatine transporter regulators can be developed to differentially modulate creatine flux in skeletal muscle (enhancing energy expenditure) and adipose tissue (amplifying futile cycling). By integrating metabolomics and proteomics, the dynamic changes of the exercise–creatine axis in various tissues can be mapped, identifying key regulatory factors for therapeutic applications. Integrating spatial transcriptomics and single‐cell metabolomics can elucidate the microenvironmental heterogeneity of creatine metabolism in adipose tissue. Utilizing CRISPR activation (CRISPRa) tools, CKB expression in adipocytes can be selectively enhanced to amplify futile cycling (thermogenesis).

Additonally, exercise can modulate the dynamic regulation of CRT and myokine–exosome interactions, optimizing the creatine metabolism network across tissues. Studies have found that acute exercise activates the AMPK/PGC‐1α signalling pathway, which may upregulate CRT expression, accelerating creatine uptake and ensuring rapid replenishment of the phosphagen system. Exercise‐induced myokines (such as irisin) and muscle‐derived exosomal miRNAs (such as miR‐27a) enhance futile creatine cycling and UCP1‐independent thermogenesis in adipose tissue. However, the mechanisms of inter‐tissue creatine transport (such as exosome shuttling and CRT isoform‐mediated flux) remain unclear. CRT deficiency induces systemic obesity, whereas creatine supplementation mitigates lipid accumulation in the liver and adipose tissue (Chen et al., 2023; Finch et al., 2024; Kreider & Stout, 2021). Over‐supplementation of creatine may downregulate CRT via negative feedback, offsetting the benefits of exercise – personalized dosing regimens are needed. Current creatine formulations lack fat specificity; nanocarrier systems or CRT agonists could improve fat targeting. Additionally, high‐resolution imaging techniques can monitor the distribution and dynamic changes of creatine in different tissues in real‐time, leading to further understanding of its physiological functions and differences under pathological conditions.

## 5 CONCLUSIONS

The skeletal muscle–adipose creatine metabolic axis updates our understanding of the metabolic health benefits of exercise. This system, by aligning the efficiency of the creatine shuttle with the activity of adipose futile cycling, establishes a barrier against lipid accumulation. Future studies should focus on conducting spatiotemporal analyses of creatine metabolism through single‐cell metabolomics to elucidate tissue‐specific dynamics; employing CRISPR‐based methodologies to selectively activate genes associated with adipose creatine metabolism, such as *CKB* and *GATM*, thereby promoting tissue‐specific thermogenesis; and devising personalized exercise and creatine supplementation protocols that accommodate the metabolic diversity inherent in obesity. This comprehensive approach not only enhances our understanding of energy balance but also highlights avenues for the creation of advanced anti‐obesity interventions. The convergence of mechanistic insights with translational advancements will be crucial in tackling the global obesity epidemic.

## AUTHOR CONTRIBUTIONS

Yuhui Su wrote the article; Na Liu, Yang Liu, Yiqun Sun, and Yike Jiao revised it. All authors have read and approved the final version of this manuscript and agree to be accountable for all aspects of the work in ensuring that questions related to the accuracy or integrity of any part of the work are appropriately investigated and resolved. All persons designated as authors qualify for authorship, and all those who qualify for authorship are listed.

## CONFLICT OF INTEREST

None declared.

## FUNDING INFORMATION

This research received no external funding.
